# Combined Effects of the *Pijolavirus UFJF_PfSW6* Phage and Sodium Hypochlorite for Reducing *Pseudomonas fluorescens* Biofilm

**DOI:** 10.3390/microorganisms12122523

**Published:** 2024-12-07

**Authors:** Matheus B. Mendes, Pedro M. P. Vidigal, Maryoris E. Soto Lopez, Humberto M. Hungaro

**Affiliations:** 1 Departamento de Ciências Farmacêuticas, Faculdade de Farmácia, Universidade Federal de Juiz de Fora (UFJF), Juiz de Fora 36036-900, MG, Brazil; matheusbragamendes@gmail.com; 2 Núcleo de Análise de Biomoléculas (NuBioMol), Campus da UFV, Universidade Federal de Viçosa (UFV), Viçosa 36570-900, MG, Brazil; pedro.vidigal@ufv.br; 3 Departamento de Ingeniería de Alimentos, Universidad de Córdoba, Montería 230002, Colombia; mesoto@correo.unicordoba.edu.co

**Keywords:** viruses, anti-biofilm, adhesion, chlorine

## Abstract

*Pseudomonas* are significant spoilage bacteria in raw milk and dairy products, primarily due to their ability to form biofilms and resist disinfection. This study explored the effects of the *UFJF_PfSW6* phage combined with sodium hypochlorite in reducing *Pseudomonas fluorescens* biofilms on stainless steel at various temperatures and ages. Biofilms were formed using *P. fluorescens* UFV 041 in UHT milk, incubated at 4 °C and 30 °C for 2 and 7 days. Two lytic phages were compared, with *UFJF_PfSW6* showing superior activity, reducing cell counts by 0.8 to 2.0 logs CFU/cm^2^ depending on conditions. Increasing the contact time of the *UFJF_PfSW6* phage from 4 to 8 h did not significantly affect the reduction in mature biofilms. The individual treatments of the phage and sodium hypochlorite (100 mg/L) reduced bacterial counts by 0.9 and 0.6 log CFU/cm^2^ at 30 °C, and 1.3 and 1.2 log CFU/cm^2^ at 4 °C, respectively. However, their sequential application achieved greater reductions, reaching 1.3 and 1.8 log CFU/cm^2^ for biofilms formed at 30 °C and 4 °C, respectively. These findings suggest a promising strategy for controlling *P. fluorescens* in the food industry. Our findings suggest that the *UFJF_PfSW6* phage combined with chlorine improves the removal of *P. fluorescens* biofilms.

## 1. Introduction

The *Pseudomonas* genus is notable for its physiological and metabolic diversity, which allows it to occupy a wide range of ecological niches [1]. These bacteria are ubiquitous, surviving in environments as varied as the atmosphere, soil, and water [1,2]. In particular, cold-adapted *Pseudomonas* species can colonize low-temperature environments, where psychrophilic and psychrotolerant species thrive [2].

The *Pseudomonas* genus comprises many species, including *P. aeruginosa*, *P. syringae*, *P. chlororaphis*, *P. putida*, and *P. fluorescens* [2]. *P. fluorescens* is a common contaminant in refrigerated foods, such as milk and dairy products [3]. These bacteria produce copious quantities of exopolymeric compounds, which are indispensable for attachment to moist surfaces and the formation of biofilms [4].

Biofilms are complex microbial communities that are protected by a polymeric extracellular matrix. This matrix provides shelter in adverse environments and guarantees nutrients necessary for the development of microorganisms [5]. In the food industry, biofilms are frequently encountered on surfaces, equipment, and pipes, presenting a significant challenge due to their difficult removal and resistance to cleaning agents [6,7]. Biofilms protect and absorb nutrients, and facilitate cell metabolic interactions [2,8]. Psychrophilic enzymes, such as phospholipases and proteases, are produced by these bacteria to maximize activity at low temperatures [2,9]. This species produces enzymes, including protease, lecithinase, and lipase, which affect raw and pasteurized milk, causing bitterness, sedimentation, and gelation problems [10].

Furthermore, pathogenic microorganisms in these biofilms increase the risk of cross-contamination, affecting food quality and safety [11]. Cross-contamination in the food production chain results in reduced shelf life and increases the risk of foodborne illnesses [12]. Biofilms have been identified as a significant contributor to negative economic impacts, particularly within the food and dairy industry [12]. In the dairy industry, biofilms can alter the organoleptic characteristics of products and reduce their shelf life. Milk is a source of nutrients that favors the development of biofilms in pipes and tanks [13]. The food industry employs stainless steel due to its corrosion resistance. However, the adherence of microorganisms to the surface and the subsequent formation of biofilms accelerates the process of steel degradation by biofouling [14,15].

Current biofilm control strategies employed in the food industry, including cleaning, disinfection, and enzyme-based detergents, are frequently utilized to control biofilms [16]. Physical methods (hot steam and ultrasonication) and chemical agents (including sodium hypochlorite) are also commonly employed to prevent biofilm formation on surfaces [17]. However, novel approaches, including bacteriocins, quorum sensing inhibitors, essential oils, and bacteriophages, are currently being investigated as potential solutions to enhance biofilm control in the food industry [16,17].

As an innovative solution to the problems caused by biofilms, bacteriophages (also known as phages) are emerging as a promising alternative. Phages are specific viruses for bacteria and are naturally present in the environment [18]. Phages have been identified as a promising alternative for controlling and eliminating biofilms in various areas [19]. Phages can readily penetrate biofilms through channels and pores that transport nutrients, degrade the exopolysaccharides matrix, reach the biofilm’s basal layer, and infect and destroy bacterial cells [20]. Currently, lytic phages are particularly efficacious in the biocontrol of food pathogens due to their low production cost and safety in food applications [21,22]. Phage cocktails, composed of multiple phages, have been successfully evaluated in the context of biofilm treatment and have demonstrated the capacity to reduce biofilms of *Listeria monocytogenes* [23,24] and *Salmonella* [25,26]. Nevertheless, certain obstacles, such as the potential emergence of bacterial resistance to phage therapy, must be addressed to achieve the eradication of biofilms [24].

The objectives of this study were (i) to compare the activities of two lytic phages against *P. fluorescens* biofilms formed under different conditions, (ii) to evaluate the influence of increased contact time on the reduction in mature biofilms by phage, and (iii) to evaluate the sequential combined effect of phage and sodium hypochlorite on reducing mature biofilms.

## 2. Materials and Methods

### 2.1. Bacterial Strain and Culture Conditions

The *P. fluorescens* UFV 041 used in this study was kindly provided by the Culture Collection of the Laboratory of Food Microbiology, Department of Microbiology, Federal University of Viçosa, Brazil. The strain was stored at −80 °C under cryoprotection (glycerol 20% *v*/*v*) until further use. The bacterium was grown in 3 mL of tryptic soy broth (TSB) (Himedia, Mumbai, India) at 30 °C for 24 h, centrifuged (5000× *g*, 5 min, 4 °C), resuspended in 0.85% (*w*/*v*) sodium chloride solution, and adjusted to an OD_600nm_ of 0.1 (10^8^ CFU/mL). Then, the cell suspension was serially diluted (10-fold) in 0.1% (*w*/*v*) peptone water until reaching 10^4^ CFU/mL for inoculation of the biofilm formation test.

### 2.2. Phages and Titration of Stock Solution

In this study, two lytic bacteriophages were used against biofilm formed by *P. fluorescens*. The UFJF_PfDIW6 and UFJF_PfSW6 phages were previously isolated from dairy industry wastewater and stream water, respectively, and characterized by our research group [27,28,29]. The UFJF_PfDIW6 phage is a member of the *Purivirus* genus proposed by our research group and recognized by ICTV, while the UFJF_PfSW6 phage is a novel species within the *Pijolavirus* genus [27,29]. Both phages were propagated and stored together with the host bacterium at −80 °C under cryoprotection (glycerol 20% *v*/*v*) according to Sambrook and Russell [30]. Suspensions of phages in SM buffer (50 mM Tris-HCl [pH 7.5], 0.1 M NaCl, 8 mM MgSO_4_ × 7H_2_O, 0.01% gelatin) were also stored at 4 °C for assays against biofilms. Before each test, phage suspensions were titrated using the drop-on-lawn technique according to Adams [31] with modifications. Briefly, 500 μL of bacterial overnight culture and 3 mL of TSB 0.7% (*w*/*v*) agar were mixed in a tube and plated into a tryptic soy agar (TSA) (Himedia, Mumbai, India). Aliquots of 20 μL of serial dilutions (10-fold) of the phage suspension were spotted on layers of overlay TSA containing the *P. fluorescens* UFV 041 (host bacterium). Plates were incubated at 30 °C for 24 h, and posteriorly, lysis plaque was counted and expressed as plaque-forming units (PFU)/mL.

### 2.3. Biofilm Formation on Stainless Steel Surface

Stainless steel coupons (SSCs—AISI #304, 10 mm × 10 mm × 1 mm) were prepared according to the method described by Parizzi et al. with some modifications [32]. Briefly, stainless steel coupons were sequentially immersed in acetone (PA, CRQ, Diadema, Brazil) for 30 min, and in sodium hydroxide solution (1 M, Neon, Suzano, Brazil) for 1 h. Afterward, they were rinsed with distilled water, suspended by a polyamide thread into a Schott flask, and autoclaved at 121 °C for 15 min.

Schott flasks containing the SSCs were added with 200 mL of UHT milk as a culture medium for biofilm formation. UHT milk was previously evaluated for aerobic mesophilic microorganism counts, as described by Laird et al., resulting in <1 CFU/mL [33]. *P. fluorescens* UFV 041 was inoculated into UHT milk to a final concentration of 10^4^ CFU/mL. Flasks were incubated at 30 °C and 4 °C to form the young biofilm (2 days) and the mature biofilm (7 days). Then, SSCs were rinsed thrice in 0.1% (*w*/*v*) peptone water for 1 min to remove planktonic cells and transferred to tubes containing 10 mL of 0.1% (*w*/*v*) peptone water. The sessile cells in the biofilms were detached from the coupons using an ultrasonic bath at 40 ± 2 kHz for 3 min. Afterwards, the obtained cell suspension was serially diluted (1:10) in 0.1% (*w*/*v*) peptone water. Bacterial counts were performed using the drop plate inoculation technique onto TSA and incubation at 30 °C for 24 h. The results were expressed in colony-forming units (CFU)/cm^2^.

### 2.4. Effects of Phage Treatments on the Removal of Biofilms

SSCs containing biofilms formed at different temperatures (30 °C and 4 °C) and incubation times (2 and 7 days) were rinsed three times successively in 0.1% (*w*/*v*) peptone water for 1 min to remove planktonic cells. Then, SSCs were individually submerged into phage suspensions, UFJF_PfDIW6 (10^8^ PFU/mL) and UFJF_PfSW6 (10^7^ PFU/mL) at 25 °C for 4 h, and sodium hypochlorite solution (100 mg/L of residual-free chlorine) at 25 °C for 10 min. Sodium hypochlorite was selected as the sanitizer for this comparative study with phages due to its widespread use in the Brazilian dairy industry, low cost, broad spectrum of activity, and ease of acquisition and handling. The residual-free chlorine concentration was measured before each treatment using an N,N′-diethyl-p-phenylenediamine (DPD) method and a Pocket Colorimeter II (Hach Company, Loveland, CO, USA). SSCs submerged in SM buffer at 25 °C for 4 h were used as a negative control. Then, all coupons were subjected to quantification of survival cells in the biofilm.

### 2.5. Extending Contact Time of Phage Treatment

The UFJF_PfSW6 phage was selected for further investigation into the effects of increasing contact time on reducing *Pseudomonas* counts in mature biofilms formed at 30 °C and 4 °C for 7 days. According to the previously established protocol, *P. fluorescens* UFV 041 was used again to form mature biofilms on SSCs. SSCs containing biofilms were rinsed thrice in 0.1% (*w*/*v*) peptone water for 1 min. These coupons were submerged in a UFJF_PfSW6 phage suspension (10^7^ PFU/mL) at 25 °C for 4 or 8 h. SSCs submerged in SM buffer at 25 °C for 8 h were used as a negative control. Then, all coupons were subjected to quantification of survival cells in the biofilm.

### 2.6. Sequential UFJF_PFSW6 Phage and Sanitizer Treatments

The combined effect of sequential treatments with phage and sanitizer on mature biofilm reduction was also investigated. Mature biofilms were formed at 30 °C and 4 °C on SSCs for 7 days as described above. Then, each coupon was rinsed thrice in 0.1% (*w*/*v*) peptone water for 1 min. SSCs were submerged in the UFJF_PfSW6 phage suspension (10^7^ PFU/mL) for 4 h, followed by a submersion in a fresh sodium hypochlorite solution (100 mg/L of residual-free chlorine) at 25 °C for 10 min. Then, all coupons were subjected to quantification of survival cells in the biofilm.

### 2.7. Quantification of Survivor Cells in Biofilm

SSCs exposed to phage treatment were rinsed thrice in 0.1% (*w*/*v*) peptone water for 1 min. SSCs exposed to sodium hypochlorite were rinsed in 0.1% (*w*/*v*) peptone water with 0.5% (*w*/*v*) sodium thiosulfate (chlorine neutralizer). All SSCs were transferred to tubes containing 10 mL of 0.1% (*w*/*v*) peptone water and subjected to an ultrasonic bath at 40 ± 2 kHz for 3 min to remove survival cells in the biofilm. Bacterial counts in the cell suspension were performed using the drop plate inoculation technique onto TSA and incubation at 30 °C for 24 h. The results were expressed in colony-forming units (CFU)/cm^2^.

### 2.8. Statistical Analysis

Data were initially assessed for normal distribution and homogeneity of residual variances to ensure adherence to analysis of variance (ANOVA) assumptions. When the data did not meet these assumptions, nonparametric tests were employed. The Kruskal–Wallis test, followed by Dunn’s multiple comparisons tests, analyzed the statistical differences among the treatments. For comparisons between two normally distributed datasets, the Student *t*-test was applied. When ANOVA indicated significant differences, Scott–Knott’s test was used to analyze the treatment groups further. The significance level was set at *p* < 0.05. GraphPad Prism (GraphPad Software, Boston, MA, USA) processed most statistical analyses, while Scott–Knott’s test was performed using the software SISVAR (SISVAR, UFLA, version 5.8, Lavras, Brazil) [34]. All the experiments were performed in triplicate.

## 3. Results

### 3.1. Biofilm Formation on SSC

**Figure 1:**
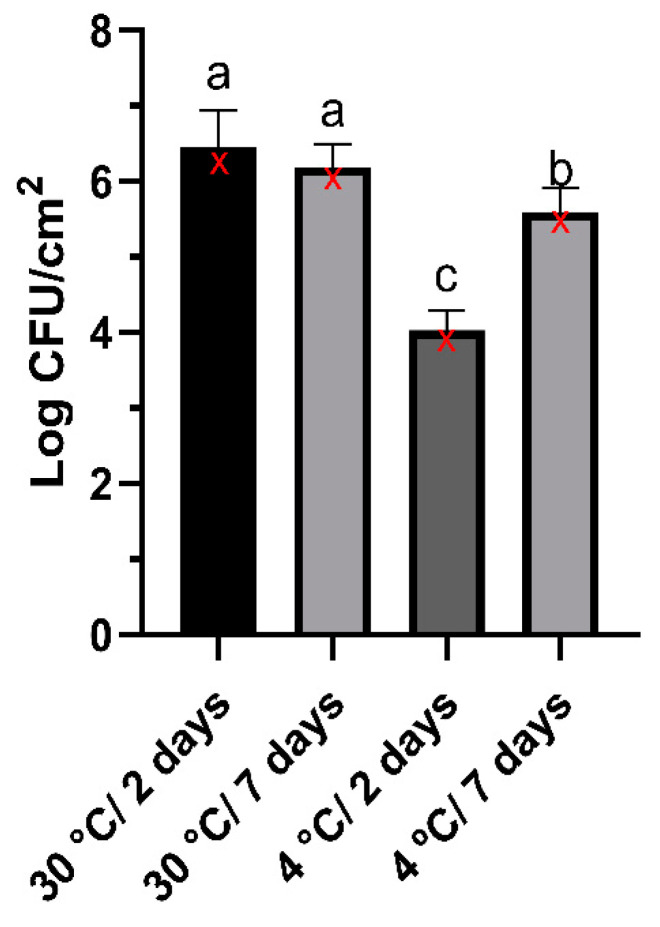
Effect of temperature and incubation time in biofilm formation on stainless steel surface by *P. fluorescens* UFV 041. Results are presented as mean ± standard deviation. Data analysis was performed using ANOVA (result = *p <* 0.0001). Means followed by the same letter do not differ statistically by the Scott–Knott test (*p* > 0.05) for each condition. Medians for each experimental condition are marked as red “X” symbols.

The count of *P. fluorescens* on the SSC surface at 30 °C was 6.45 ± 0.48 log CFU/cm^2^ after 2 days and remained relatively constant at 6.19 ± 0.32 log CFU/cm^2^ after 7 days. In contrast, the bacterial count was 4.03 ± 0.26 log CFU/cm^2^ after 2 days at 4 °C, increasing to 5.59 ± 0.32 log CFU/cm^2^ after 7 days of incubation. The *P. fluorescens* count on the SSC surface was higher at 30 °C than at 4 °C regardless of the incubation time (Figure 1).

### 3.2. Effects of Individual Phages and Sanitizer on Biofilms Removal

**Figure 2:**
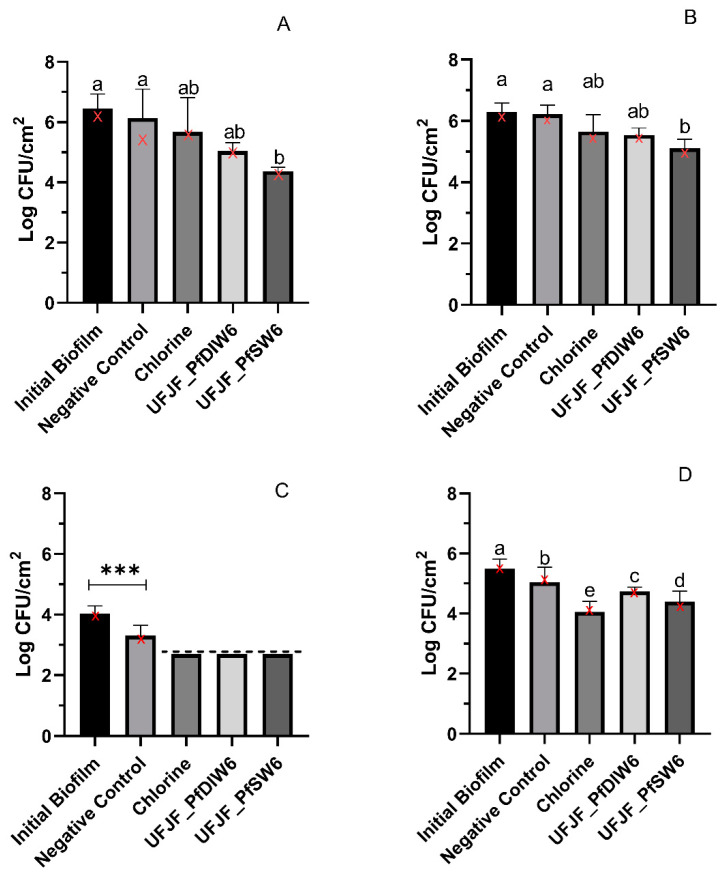
Effect of UFJF_PfDIW6, UFJF_PfSW6, and sodium hypochlorite treatments on *P. fluorescens* counts in biofilms formed at 30 °C (**A**,**B**) and 4 °C (**C**,**D**) for 2 days (**A**,**C**) and 7 days (**B**,**D**). Initial biofilm—bacterial count in the biofilm before treatments. Negative control—bacterial count after submersion in SM buffer for 4 h. The dashed line represents the detection limit of the experiment (>2.70 log CFU/cm^2^). Results are presented as mean ± standard deviation. The Kruskal–Wallis test followed by Dunn’s multiple comparisons test were used for data analysis in (**A**) (result—*p* = 0.0008) and (**B**) (result—*p* = 0.0003). Results in (**C**) were compared using the Student *t*-test (*** *p* = 0.0004), as the remaining treatment results were below the detection limit. ANOVA followed by the Scott–Knott test was performed for data analysis in (**D**) (result—*p* < 0.0001). Results followed by the same letter do not differ statistically (*p* > 0.05) for each incubation period. Medians for each experimental condition are marked as red “X” symbols.

The phages individually applied at 25 °C for 4 h reduced the bacterial counts in both biofilm grown at 30 °C for 2 days (young) and biofilm grown at 30 °C for 7 days (mature). Although UFJF_PfDIW6 achieved reductions of 1.42 log CFU/cm^2^ and 0.75 log CFU/cm^2^ in young and mature biofilms, respectively, they did not statistically differ, by Dunn’s test, from the counts observed in the initial biofilm and negative control. In contrast, UFJF_PfSW6 exhibited statistically significant reductions under both conditions, achieving reductions of 2.08 log CFU/cm^2^ and 1.17 log CFU/cm^2^, respectively. The efficacy of both phages in reducing bacterial count was diminished by approximately 50% against mature biofilms compared to young biofilms. The treatment of biofilms with 100 mg/L of residual-free chlorine at 25 °C for 10 min was minimally effective in removing bacterial cells, reducing <1.0 log CFU/cm^2^ in both young and mature biofilms (Figure 2A,B).

Regarding the biofilms grown at 4 °C, the phages and sanitizer demonstrated reductions in bacterial count >1.3 log CFU/cm^2^ when applied against young biofilms, with cell counts falling below the limit of quantification after decontamination treatments (Figure 2C). As observed with biofilms grown at 30 °C, the UFJF_PfSW6 phage demonstrated a more significant reduction in the bacterial count of mature biofilms formed at 4 °C, reducing the count by 1.10 log CFU/cm^2^, compared to the UFJF_PfDIW6 phage, which reduced it by 0.76 log CFU/cm^2^ (Figure 2D). The growth temperature of the biofilms (30 °C or 4 °C) did not influence the reduction in bacterial count by either of the phages evaluated (*p* > 0.05). However, chlorine’s bacterial count reduction activity was more significant in biofilms formed at 4 °C/7 days (1.44 log CFU/cm^2^) than those formed at 30 °C/7days (0.63 log CFU/cm^2^).

### 3.3. Effect of Extending UFJF_PFSW6 Phage Treatment on Biofilm Removal

**Figure 3:**
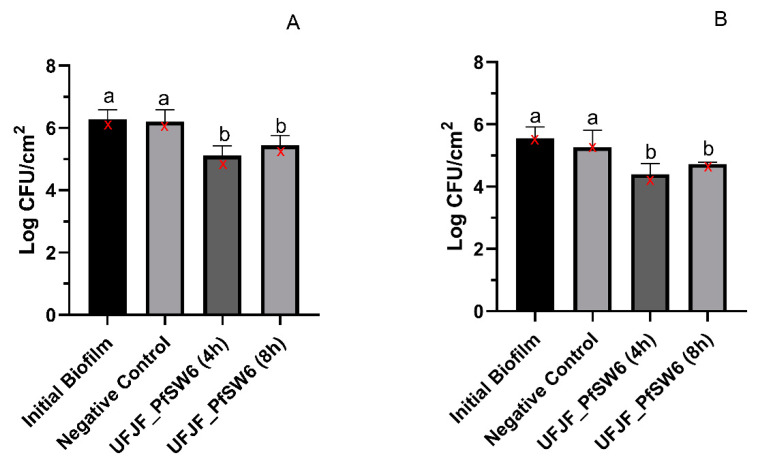
Effect of extending contact time of phage treatment on reducing bacterial counts in biofilms formed at 30 °C (**A**) and 4 °C (**B**) after 7 days. Initial biofilm—bacterial count in the biofilm before treatments. Negative control—bacterial count after submersion in SM buffer for 8 h. Results are presented as mean ± standard deviation. ANOVA was performed for data analysis in (**A**) and (**B**). Means followed by the same letter do not differ statistically by the Scott–Knott test (*p* > 0.05) for each condition. Medians for each experimental condition are marked as red “X” symbols.

The influence of extending the contact time of the bacteriophage treatment from 4 to 8 h on the reduction in the bacterial count was investigated against mature biofilms formed at 30 °C and 4 °C for seven days. The UFJF_PfSW6 phage was chosen for this assay due to its greater biofilm reduction efficacy than UFJF_PfDIW6 under both formation biofilm conditions. Increasing the contact time of the biofilm with the phage from 4 to 8 h did not significantly influence the reduction in bacterial count (*p* > 0.05). The UFJF_PfSW6 phage reduced the bacterial count in biofilms formed at 30 °C by 1.16 log CFU/cm^2^ and 0.83 log CFU/cm^2^, after 4 h and 8 h of contact, respectively (Figure 3A). Nearly identical results were observed against biofilms formed at 4 °C, with reductions in the bacterial count of 1.16 log CFU/cm^2^ and 0.82 log CFU/cm^2^ for 4 and 8 h of contact with the phage, respectively (Figure 3B).

### 3.4. Effect of Sequential Treatment with UFJF_PFSW6 Phage and Sodium Hypochlorite on Biofilm Removal

**Figure 4:**
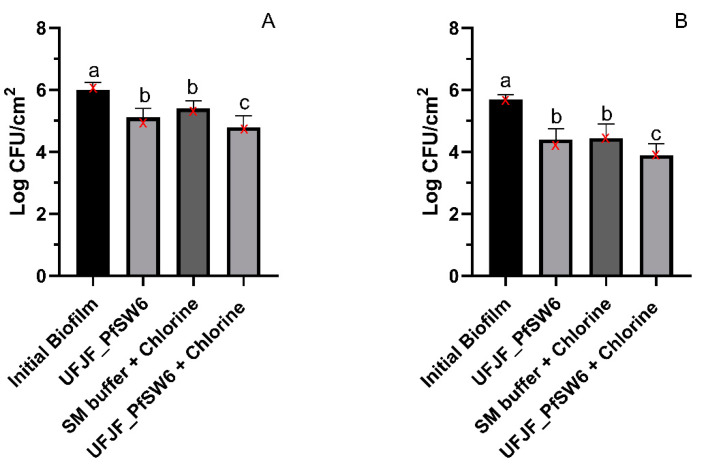
Effect of sequential treatment with UFJF_PfSW6 phage (4 h) followed by sanitizer (100 ppm for 10 min) on the reduction in bacterial counts in biofilms formed at 30 °C (**A**) and 4 °C (**B**) for 7 days. Initial biofilm—bacterial count in the biofilm before treatments. Results are presented as mean ± standard deviation. ANOVA was performed for data analysis in (**A**) and (**B**). Means followed by the same letter do not differ statistically by the Scott–Knott test (*p* > 0.05) for each condition. Medians for each experimental condition are marked as red “X” symbols.

The sequential treatment with the phage and sanitizer reduced bacterial counts in biofilms compared to individual treatments. The UFJF_PfSW6 phage reduced 0.88 log CFU/cm^2^, while chlorine treatment alone reduced 0.59 log CFU/cm^2^ in the mature biofilm formed at 30 °C. The combination of phage and chlorine reduced 1.20 log CFU/cm^2^ (Figure 4A). Similar results were observed for mature biofilms formed at 4 °C, wherein the UFJF_PfSW6 phage, chlorine, and combined treatment reduced the bacterial counts at 1.30 log CFU/cm^2^, 1.25 log CFU/cm^2^ and 1.81 log CFU/cm^2^, respectively (Figure 4B). These findings suggest a combined effect between the phage and sanitizer in mature biofilm removal at both temperatures.

## 4. Discussion

In this study, we used a strain of *P. fluorescens* (UFV 041) previously identified as a spoilage bacterium of milk and dairy products [35,36]. This bacterium can degrade milk proteins even at temperatures such as 4 °C, 7 °C, and 10 °C [37,38]. Here, we demonstrate that this bacterium can also adhere to the surface of stainless steel and that temperature and incubation time influence the number of adhered cells in the biofilms. The number of sessile cells adhered to biofilms of *P. fluorescens* UFV 041 formed at the optimal growth temperature was higher than that of biofilms formed at refrigeration temperature. In addition, the extended incubation period resulted in more cells adhering to biofilms grown at refrigeration temperature. Several factors can influence biofilm formation, including inherent characteristics of the bacterial strain, surface properties (roughness and charge), nutrient availability and type, and temperature [39]. Studies have also shown that raising the incubation temperature closer to the optimal level significantly increases the number of cells adhering to *P. fluorescens* biofilms, reaching counts 1–2 logs higher than those grown at lower temperatures [4,40]. The incubation duration significantly influences the number of cells present within biofilms. Sillankorva et al. observed an increase of 6 to 8 logs in the cell count of *P. fluorescens* biofilms cultivated at 30 °C by extending the incubation period from 1 to 7 days [41,42]. However, strains can vary significantly between biofilm formation, composition, and architecture. Some strains exhibit greater adhesion capacity and produce adhesion factors like exopolysaccharides, contributing to their environmental persistence [43]. Our findings show that *P. fluorescens* UFV 041 is a promising candidate for studies of the bacteriophages’ anti-biofilm effect. Its ability to adhere at 30 °C and 4 °C, combined with its known spoilage potential, makes it a suitable target for such studies.

We first compared the individual effects of two phage species, isolated and characterized previously by our research group, against *P. fluorescens* UFV 041 biofilms formed at 30 °C and 4 °C by 2 days and 7 days. Both phages effectively reduced *Pseudomonas* counts in biofilms formed only at 4 °C. Notably, the efficacy of the *UFJF_PfSW6* phage was not influenced by the temperature at which the biofilms were formed. However, the age of the biofilm affected and led to an approximately 50% decrease in cell reduction in mature biofilms. Biofilm composition and matrix structure can hinder phage efficacy by blocking penetration and limiting access to deep-lying bacterial cells. Mature biofilms typically exhibit a more complex three-dimensional structure with a more significant number of adhered cell layers and a higher quantity of exopolysaccharides (EPSs), which form a physical barrier to the penetration of external agents, thus reducing phage activity [44,45,46,47]. Phages are less effective at reducing *L. monocytogenes* in mature biofilms than in young biofilms [23,48]. On the other hand, Olszak et al. demonstrated that phage PA5oct primarily affects mature biofilms of *P. aeruginosa* cultivated for 72 h, contradicting the general notion that phages exhibit higher activity against younger biofilms [49]. The efficacy of phages against mature biofilms appears to be highly dependent on phage-specific characteristics, such as the production of polysaccharide-degrading enzymes capable of penetrating denser biofilms and the ability to infect cells at various physiological stages, each expressing distinct receptor structures [50,51].

In our study, using bacteriophages against biofilms of *P. fluorescens* resulted in a reduction in cell numbers ranging from 0.8 to 2.0 logs CFU/cm^2^, depending on the phage type and biofilm formation conditions. The *Pijolavirus UFJF_PfSW6* exhibited higher activity against biofilms than the *Purivirus UFJF_PfDIW6* in all experimental conditions. The efficacy of phage action depends on the specific interaction between the phage and the bacterial strain involved. Phages have anti-biofilm mechanisms, such as targeting exopolysaccharides with tail fibers and depolymerases and lysing cells through infection and endolysins, leading to biofilm disruption and cell inactivation [52,53,54,55]. Differences between the two phages analyzed in this study, mainly related to infection cycle parameters, may explain the different anti-biofilm activity results. The *Pijolavirus UFJF_PfSW6* showed a latent period of 25 min, while that of the *Purivirus UFJF_PfDIW6* was 115 min [28]. Other studies applying bacteriophages have also observed variations in the reduction in cell numbers in biofilms formed by *Pseudomonas*. For example, Sillankorva et al. demonstrated that the φIBB-PF7A phage can significantly reduce *P. fluorescens* biofilm (3–5 logs in 4 h) and adhered cell numbers (3–3.5 logs CFU/cm^2^ in 2 h), both on stainless steel surfaces [41,42]. On the other hand, Magin et al. reported reductions comparable to those observed in the present study [56]. The phages LUZ7 and 14.1 used by these authors demonstrated efficacy in reducing two distinct biofilm-forming strains of *P. aeruginosa*, resulting in reductions of 0.72 to 1.5 log CFU/cm^2^ and 1.2 to 1.7 log CFU/cm^2^, respectively [56].

When comparing the effect of phages and sanitizer, phages had an equivalent or superior effect to sodium hypochlorite against both young and mature biofilms cultivated at 30 °C. However, the sanitizer significantly reduced sessile cells in mature biofilms formed at 4 °C. Similar results were observed by Yang et al. when comparing the effect of phages and chlorine against *Salmonella* Enteritidis biofilms cultivated at 25 °C and 4 °C [57]. At 4 °C, biofilms tend to form more easily when nutrients are limited, but their resistance to chlorine varies depending on the bacterial strain involved [57].

Given the comparative results of the two phages, we chose the *Pijolavirus UFJF_PfSW6* to determine if extending the contact time would lead to a more significant reduction in mature biofilm cells. Nevertheless, increasing contact time from 4 h to 8 h did not enhance phage efficacy against mature biofilms. Other studies have also investigated the effectiveness of prolonged phage exposure in reducing biofilms. Yuan et al. demonstrated that extending the phage vB_PaeM_LS1 contact time from 4 h to 8 h resulted in a more significant reduction in *P. aeruginosa* cells in biofilm, from 1.2 log CFU/mL to 2.5 log CFU/mL, respectively [58]. However, they observed that increasing the exposure time to 12 h and 24 h did not lead to significant differences in biofilm cell reduction. Wang et al. also demonstrated a decrease in *Cronobacter sakazakii* biofilm cell counts (2.79 log CFU/cm^2^) within the first 2 h of exposure to phage JK004 [59]. However, extending the contact time to 6 h did not enhance the initial reduction. The efficacy of phages against biofilms is contingent upon several factors, including the concentration of phages, the surface properties of the biofilm, and the presence of bacterial resistance mechanisms [60]. Studies have indicated that biofilm persistence is due to some factors, including the slow metabolism of deep-layer cells, the presence of debris containing phage receptors, and the development of phage resistance [24,61].

To further enhance the reduction in cells in mature *P. fluorescens* biofilms, we assessed the combined sequential effect of *UFJF_PfSW6* phage treatment followed by sodium hypochlorite sanitization. Our data demonstrated that this sequential combined treatment resulted in a more significant reduction in biofilm cells than individual treatments. As previously reported, polysaccharide-degrading enzymes or structures with this activity on the phage tail fibers can degrade the biofilm matrix, facilitating subsequent penetration of the sanitizer into deeper biofilm layers. In addition, these enzymes or structures with hydrolytic activity can work synergistically with the sanitizer on the bacterial cell wall, making it more challenging for bacteria to repair the damaged cell envelope [61]. Other authors also evaluated the combined effect of phages and antimicrobials against biofilms of various bacteria, including *Escherichia coli* O157:H7 [60], *P. aeruginosa* [62], *S.* Typhimurium [63], and *Staphylococcus aureus* [61]. The effectiveness of this combined treatment appears to depend on the specific characteristics of both the antimicrobial compound (e.g., structure and mode of action) and the phage (e.g., ability to degrade EPS), as well as their synergistic activity and inherent characteristics of resistance of the bacteria in the biofilm. Zhang et al. demonstrated that sequential treatment of *E. coli* O157:H7 biofilms on lettuce with phage FP43 for 24 h and chlorine (40 mg/L for 5 min) resulted in more significant reductions (2.3 to 2.4 log) than when either phage (1.2 log) or chlorine (1.5 log) was used alone [60]. A similar effect was obtained when Stachler et al. sequentially combined two phages (JG004 and P1 for 4 h) and sodium hypochlorite (200 ppm for 10 min) against *P. aeruginosa* biofilms on a plastic surface, achieving reductions in biofilm cells exceeding 3 logs [62].

On the other hand, Yüksel et al. reported only reductions of up to 70% in the biomass of mature *S.* Typhimurium biofilms when they applied a combined treatment of phage P22 (10^1^ to10^9^ PFU/mL), EDTA (2.5 to 320 mM), and nisin (150 to 19,200 µg/mL) [63]. Duc et al. also observed no significant combined effect between nisin (100 IU/mL) and SA46-CTH2 phage against *S. aureus* biofilms [61]. The combination of antibiotics and phages has also proven to be a promising treatment for biofilms of clinically relevant bacteria. Vera-Mansilla et al. observed that phages enhance the efficacy of ampicillin against biofilm-associated uropathogenic *E. coli* [64]. Furthermore, a phage-derived lysin has been suggested to eliminate persister cells and biofilms [65]. Zhang et al. showed that sequential application of LysSTG2, an endolysin from *Salmonella*-lytic bacteriophage STG2, and acidic hypochlorous water containing 40 ppm chlorine resulted in a synergistic effect, leading to a reduction in biofilm cell viability of more than 99.99% [66]. As observed above, the effectiveness of combining phage and antimicrobial treatments in reducing biofilm cell numbers varies greatly. Complete biofilm eradication, however, remains a challenge and a persistent issue in various sectors, such as the food industry.

## 5. Conclusions

The *Pijolavirus UFJF_PfSW6* reduced the count of *P. fluorescens* in both young and mature biofilms formed at 30 °C and 4 °C. Its efficacy was not influenced by the temperature at which the biofilms were formed. However, the age of the biofilm did affect its efficacy, leading to a decrease in cell reduction in mature biofilms. The *UFJF_PfSW6* phage exhibited more activity against biofilms than the *UFJF_PfDIW6* phage in all experimental conditions. Nevertheless, increasing the contact time did not enhance the efficacy of the *UFJF_PfSW6* phage in reducing cells in mature biofilm. The combination of phage and sodium hypochlorite in a sequential treatment increased the reduction in *P. fluorescens* cells in mature biofilm compared to individual treatments. Although this combination could not wholly eradicate *P. fluorescens* biofilm, it potentiated the action of chlorine, a sanitizer commonly used in the food industry. Overall, our findings support the potential of using phages and their combination with sanitizers to improve the inactivation of cells in mature biofilm, providing a potential alternative for addressing this problem in the food industry. 

## Data Availability

The original contributions presented in the study are included in the article; further inquiries can be directed to the corresponding author.
